# New insights into the role of tetraspanin 6, 7, and 8 in physiology and pathology

**DOI:** 10.1002/cam4.7390

**Published:** 2024-07-19

**Authors:** Monika Mrozowska, Tomasz Górnicki, Mateusz Olbromski, Aleksandra Izabela Partyńska, Piotr Dzięgiel, Agnieszka Rusak

**Affiliations:** ^1^ Division of Histology and Embryology, Department of Human Morphology and Embryology Wroclaw Medical University Wroclaw Poland; ^2^ Department of Human Biology, Faculty of Physiotherapy Wroclaw University of Health and Sport Sciences Wroclaw Poland

**Keywords:** cancer, carcinogenesis, tetraspanins, *TPSAN7*, *TSPAN*, *TSPAN6*, *TSPAN7*

## Abstract

**Background:**

The tetraspanin (TSPAN) family comprises 33 membrane receptors involved in various physiological processes in humans. Tetrasapanins are surface proteins expressed in cells of various organisms. They are localised to the cell membrane by four transmembrane domains (TM4SF). These domains bind several cell surface receptors and signalling proteins to tetraspanin‐enriched lipid microdomains (TERM or TEM). Tetraspanins play a critical role in anchoring many proteins. They also act as a scaffold for cell signalling proteins.

**Aim:**

To summarise how tetraspanins 6, 7 and 8 contribute to the carcinogenesis process in different types of cancer.

**Methods:**

To provide a comprehensive review of the role of tetraspanins 6, 7 and 8 in cancer biology, we conducted a thorough search in PubMed, Embase and performed manual search of reference list to collect and extract data.

**Discussion:**

The assembly of tetraspanins covers an area of approximately 100–400 nm. Tetraspanins are involved in various biological processes such as membrane fusion, aggregation, proliferation, adhesion, cell migration and differentiation. They can also regulate integrins, cell surface receptors and signalling molecules. Tetraspanins form direct bonds with proteins and other members of the tetraspanin family, forming a hierarchical network of interactions and are thought to be involved in cell and membrane compartmentalisation. Tetraspanins have been implicated in cancer progression and have been shown to have multiple binding partners and to promote cancer progression and metastasis. Clinical studies have documented a correlation between the level of tetraspanin expression and the prediction of cancer progression, including breast and lung cancer.

**Conclusions:**

Tetraspanins are understudied in almost all cell types and their functions are not clearly defined. Fortunately, it has been possible to identify the basic mechanisms underlying the biological role of these proteins. Therefore, the purpose of this review is to describe the roles of tetraspanins 6, 7 and 8.

## 1 INTRODUCTION

The tetraspanin family (TSPAN) comprises 33 membrane receptors involved in various physiological processes in humans. Tetraspanins are surface proteins that are expressed in cells of various organisms. Localization in a cell membrane is guaranteed by four transmembrane domains (TM4SF).^1^, ^2^ These domains bind multiple cell surface receptors and signaling proteins to lipid microdomains enriched with tetraspanins (TERM or TEM).^1^, ^3^, ^4^ Tetraspanins play a crucial role in anchoring numerous proteins. Moreover, they act as scaffold or cell signaling proteins.^5^ The assemblage of tetraspanins covers an area of approximately 100–400 nm^2^.^6^, ^7^, ^8^ Tetraspanins are involved in various biological processes, such as membrane fusion, aggregation, proliferation,^9^ adhesion, cell migration, and differentiation.^10^ They can also regulate integrins, cell surface receptors, and signaling molecules. Tetraspanins form direct bonds with proteins and other tetraspanin‐family members, creating a hierarchical network of interactions and are thought to be involved in compartmentalization of cells and membranes.^1^ The role of tetraspanins was demonstrated in cancer progression, and they have been shown to have multiple binding partners and promote cancer progression and metastasis. Clinical studies have documented a correlation between the level of tetraspanin expression and prediction of cancer progression, including breast and lung cancers. Tetraspanins are understudied in almost all cell types and their functions are not clearly defined. Fortunately, it was possible to identify the basic mechanisms underlying the biological role of these proteins. Therefore, the purpose of this review is to describe the roles of tetraspanins 6, 7, and 8.

## 2 STRUCTURE OF TETRASPANINS

**FIGURE 1:**
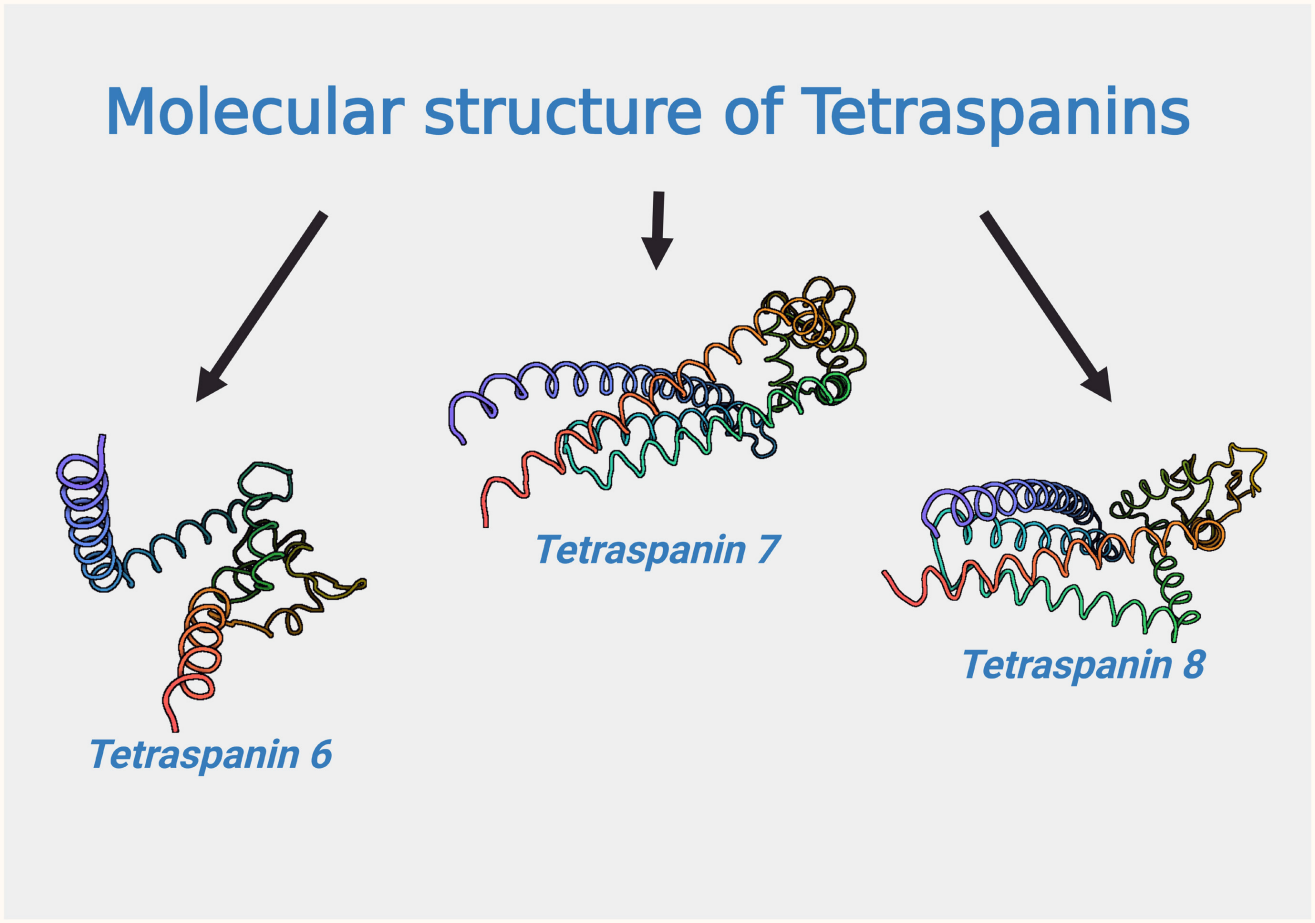
Predicted molecular structures of tetraspanins 6,7, and 8 based on AlphaFold structure prediction. The predicted alpha structures, which are conserved in all tetraspanins and facilitate homodimerization, are depicted. Variations in molecular structure among tetraspanin 6, 7, and 8 are illustrated. These models unveil characteristic loops responsible for interactions critical to the biological functions of these proteins.

Tetraspanins are made up of five different regions that combine structural features with specific functions. The predicted alpha structures are shown in Figure 1.^11^ Conserved region mediates homodimerization. The conserved domain consists of three alpha helices, whereas the variable domain is unique to each tetraspanin. A variable region located within a large extracellular loop mediates specific interactions with other proteins. The transmembrane region contains four transmembrane (TM) domains, which are likely sites of intra‐ and intermolecular interactions. These interactions are crucial for the assembly of a network of membrane proteins associated with tetraspanins, known as the ‘tetraspanin network’.^12^ The TM domains (TM1‐TM4) are bound by two extracellular regions, that is, a small loop and a large loop. The extracellular loops (ECs) are divided into two parts: EC1 and EC2. EC1 is composed of 20–28 amino acids and is located between TM1 and TM2, meanwhile EC2 contains 76–131 amino acids and is located between TM3 and TM4. EC2 is responsible for heterophilic interactions with other proteins and for the specificity of these interactions. In addition, the Cys‐Cys‐Gly amino acid motif is formed within EC2.^2^, ^13^, ^14^, ^15^, ^16^ The extracellular domain of tetraspanins consists of conserved and variable domains. EC2 domain is located above the cavity and is formed by four transmembrane domains.^17^ The N‐ and C‐termini of tetraspanins are found on the intracellular side of the cell membrane. Tetraspanins are mostly glycosylated, which is reflected by their variable molecular weights of 20–50 kDa.

## 3 CELLULAR LOCALIZATION

Tetraspanins are located in the cell membrane and act as scaffold proteins that anchor many other proteins.^18^ Tetraspanins are also thought to be involved in the formation of multimeric complexes in cell membranes. Increasing evidence suggests that tetraspanins play a key role in the regulation of the transport and function of other membrane proteins. Tetraspanins interact with each other or other membrane proteins to form tetraspanin‐enriched microdomains. They play a crucial role in development, pathogenesis, and immune response by facilitating cell–cell adhesion and fusion, ligand binding, and intracellular transport.^19^ Tetraspanins have no known adhesion ligands or catalytic activities. However, they function as molecular scaffolds that facilitate the organization of proteins into well‐structured microdomains consisting of adhesion, signaling, and adaptor proteins.^2^ Tetraspanins interact with other proteins through different regions such as the transmembrane domain or the EC2 loop, and these interactions can occur through EC2 loop, which confers specificity.^20^ Recent studies have shown that tetraspanins could affect migration by influencing adhesion and signaling via integrins, the primary adhesion receptors for extracellular matrix proteins such as collagens and laminins. Although the role of tetraspanins in carcinogenesis is not clear, recent evidence suggests that they play an important role in the proliferation of cancer cells, thereby influencing their growth at their site of origin. Their unique ability to control a broad spectrum of biological functions makes them an interesting target for research.

## 4 BETWEEN PHYSIOLOGY AND PATHOLOGY

Tetraspanin 6 (also known as T245/TM4SF6) and tetraspanin 7 (also known as TALLA‐1/TM4SF2/A15/CD231) are closely related homologs expressed in various tissues. It is worth noting that *TSPAN6* and *TSPAN7* are the only *TSPAN* genes located on the X chromosome, the long arm (Xq22.1) and short arm (Xp11.4), respectively.^21^ *TSPAN6* mRNA has been detected in various normal epithelial tissues.^22^ Tetraspanin 6 is associated with epilepsy and mental retardation, whereas tetraspanin 7 expression is restricted to the central nervous system (CNS), lungs, and endocrine organs, such as pancreatic islets and pituitary gland.^23^ However, it is noteworthy that primary tetraspanin 7 was initially identified as a novel surface marker for T‐cell acute lymphoblastic leukemia.^24^

*TSPAN7* is highly expressed in a brain and its expression can be inactivated either by chromosomal translocation (X;2) or by a point mutation in the EC2 loop (P172H), resulting in intellectual disability (ID).^25^ This may be due to the impaired ability of the actin cytoskeleton to stimulate neurite outgrowth. Tetraspanin 7 is involved in neuronal morphogenesis and regulates synaptic transmission, particularly in cortical and hippocampal neurons. *TSPAN7* mRNA level increases after treatment with kainic acid, suggesting its a role in signaling pathways that are important for synaptic transmission and neuronal plasticity.^26^, ^27^ Tetraspanin 7 is associated with various neurological disorders, including autism spectrum disorder (ASD), and published data showed that expression of this protein was reduced in the brain specimens of patients diagnosed with ASD, Huntington's disease (HD), Parkinson's disease (PD), and Alzheimer's disease (AD).^28^ The Tetraspanin 7 /β1 integrin/FAK/SRC pathway has been identified as a novel signaling pathway involved in the pathogenesis of ASD and ID. Furthermore, research has shown that tetraspanin 7 deficiency alters synaptic structure, impairs learning and weakens memory.^29^ Recent studies have shown that tetraspanin 7 affects dendritic spine formation, glutamatergic synaptic transmission, and neuronal plasticity. Therefore, this protein is associated with neuropsychiatric disorders such as HD,^25^ fragile X syndrome, and myotonic dystrophy. Tetraspanin 7 plays a crucial role in the regulation of bone resorption by osteoclasts and cytoskeletal remodeling in vitro, as previously described by Kwon et al.^30^ It is also involved in the formation of sealing zones and bone resorption activity of osteoclasts. In addition, Tetraspanin 6 and Tetraspanin 7 were shown to interact with glutamate receptors, highlighting the importance of individual tetraspanins in critical processes in the CNS. Tetraspanin 7 mutations are associated with ID, whereas tetraspanin 6 overexpression is linked to AD.^31^ Tetraspanin 6 levels are elevated in the brains of patients suffering from AD, and its overexpression in cells affects amyloid precursor protein (APP) metabolism.

Recent studies have also shown that tetraspanin 7 plays a crucial role in the immune system of patients with type 1 diabetes.^32^, ^33^, ^34^ This tetraspanin assists T and B cells in epitope identification. It has been suggested that tetraspanin 7 may serve as an autoantigen in diabetes, making it useful for diabetes prediction and immunotherapy.^32^ Tetraspanin 7 is a surface marker of mature pancreatic endocrine cells^35^ and is present in the insulin‐containing granules of pancreatic β‐cells and glucagon‐producing α‐cells^36^ and also regulates Ca^2+^‐dependent exocytosis in β‐cells.

The functions of tetraspanin 6 and 7 were discovered in the late 20th century, but are still not fully understood. Recent scientific research has provided increasing evidence for the specific mechanisms of action of tetraspanins in carcinogenesis and metastasis. Tetraspanin 8 (also known as CO‐029/TM4SF3) is mainly found in the gastrointestinal tract, particularly in the epithelium of the colon and stomach.^36^ It has been shown to be overexpressed in human glioma cells. In addition, tetraspanin 8 is present in vascular smooth muscle cells and tubular cells in normal kidney.^37^ Nuclear localization of tetraspanin 8 was observed in several cell lines, including SW1990 (pancreatic cancer), MDA‐MB‐231 (breast cancer), SW620 (colon cancer), HGC‐27 and AGS (stomach cancer), and also U‐87 MG and U251 MG (glioblastoma cell lines). Tetraspanin 8 is a molecular regulator^18^ involved in tissue differentiation,^6^ and cancer cell metastasis.^38^ In addition, tetraspanin 8 regulates proliferation, motility, and invasiveness of cancer cells.^39^

## 5 CARCINOGENESIS

### 5.1 Tetraspanin 6

In fact, there is limited knowledge about the role of tetraspanin 6 in carcinogenesis, and this has only been obtained in the last 3 years. Current research suggests that tetraspanin 6 may have anticancer effects and may play a role in the transformation and progression of colorectal, lung, pancreatic, and breast cancers.^21^ Studies carried out in an early stage colorectal cancer murine model have suggested that suppression of tetraspanin 6 expression increases the incidence and size of adenomas. In addition, decreased expression of tetraspanin 6 was associated with a poorer prognosis, whereas high expression was associated with a better response to cetuximab‐based EGFR‐targeted therapy. The proposed mechanism for the role of tetraspanin 6 in colorectal cancer progression includes the activation of the EGF‐dependent signaling pathway through loss of tetraspanin 6 expression. This activation leads to an increase in the expression of the transmembrane form of TGFα. Syntein 1 may regulate this signaling pathway as it can bind both tetraspanin 6 and the transmembrane form of TGFα.^40^ Therefore, research suggests that tetraspanin 6 can inhibit the epithelial‐mesenchymal transition (EMT).^41^

Recently, studies performed in mouse models have indicated that tetraspanin 6 acts as a tumor suppressor protein in pancreatic and lung cancers. Tetraspanin 6 is thought to interact with EGFR and inhibit the EGFR‐RAS‐ERK signaling pathway, thereby promoting the epithelial cell phenotype. It is important to note that these findings are based on mouse models and further research is needed to confirm their applicability to humans. In addition, decreased tetraspanin 6 expression is associated with shorter survival in patients with pancreatic or lung cancer.^41^ However, the role of tetraspanin 6 is not limited to cancer cells. In breast cancer, tetraspanin 6 has been shown to possibly enhance the invasion of B lymphocytes into the tumor. Tetraspanin 6 can also increase the concentration of LXR receptor ligands in extracellular vesicles (EVs), which stimulates the migration of B lymphocytes into the tumor microenvironment. This effect may strengthen the immune response against tumor cells.^42^

### 5.2 Tetraspanin 7

Role of tetraspanin 7 in carcinogenesis has not been fully understood yet. Primary, this protein was identified in 1995 by Takagi and co‐workers as a surface marker on T‐cells in acute leukemia.^24^ However, the role of tetraspanin 7 in this disease is not fully elucidated. However, some studies have suggested that it may play a role in the development and progression of various types of cancers. For example, a study concerning multiple myeloma found that overexpression of tetraspanin 7 reduced tumor size in mouse model but did not affect cell proliferation in an in vitro model.^43^

In silico analysis, conducted with machine learning modeling, indicates that the expression level of tetranspanin 7 may predict the risk of meningioma recurrence. This model may be used to indicate the need for the adjuvant radiotherapy for meningioma patients.^44^ Tetraspanin 7 is also thought to play a role in bladder cancer progression. Researchers have found decreased *TSPAN7* mRNA level in clinical cases, and a decrease in tetraspanin 7 expression was found to correlate with a poorer prognosis. Consist with these findings, in vitro studies and a mouse model have demonstrated the potential mechanisms of action of tetraspanin 7 as an anticancer protein. Tetraspanin 7 is thought to have a proapoptotic function by affecting BAX proteins and caspase 3. In addition, overexpression of tetraspanin 7 inhibits the growth of bladder cancer cells via the PTEN/PI3K/AKT pathway.^45^

Tetraspanin 7 also plays a unique role in osteosarcoma. According to available data, tetraspanin 7 is highly expressed in tissues from patients and commercially available osteosarcoma cell lines. Functional studies confirmed that inhibition of tetraspanin 7 expression stopped tumor growth and the EMT process, whereas overexpression of tetraspanin 7 had the opposite effect. Mechanistically, interaction of tetraspanin 7 with integrin β1 leads to activation of the FAK‐Src‐Ras‐ERK1/2 signaling cascade.^3^

## 6 TETRASPANIN 8

Of all the tetraspanins described in this review, tetraspanin 8 is currently the best‐studied one. Its role has been postulated in various types of cancers, suggesting many potential mechanisms of action. Therefore, it is a potential target for diagnostics and cancer therapy.

Increased expression of tetraspanin 8 was detected in malignant gliomas compared with control tissues. This study found a correlation between the expression level of tetraspanin 8 and the degree of malignancy of the tumor. Suppression of tetraspanin 8 expression led to reduced proliferation and migration capacity of glioma cells and increased the efficacy of temozolomide (TMZ) in vitro.^46^ The involvement of tetraspanin 8 in the pathogenesis of gliomas may occur through the activation of FAK kinase.^47^ Tetraspanin 8 is thought to influence the migration of malignant glioma cells by forming a complex with α3 integrin and Rictor protein. This complex plays a key role in the formation of the mTORC2 complex (mammalian target of rapamycin), which modulates cytoskeletal actin filaments via Cα‐kinase and AKT.^48^ The involvement of tetraspanin 8 in the pathogenesis of gliomas is suggested by the fact that its expression is reduced by AMP kinase whereas activation of AMP kinase leads to inhibition of cancer cell growth.^46^

Up‐to‐date analyses indicate that the expression of tetraspanin 8 was increased in gastric cancer cells compared with healthy tissues.^49^ The expression of tetraspanin 8 correlated positively with several clinicopathological features such as stage, status of lymph node invasion, and degree of tumor differentiation. In addition, higher expression of tetraspanin 8 correlates with a poorer prognosis.^50^ In vitro studies have shown that overexpression of tetraspanin 8 promotes the ability of gastric cancer cells to invade and proliferate, whereas suppression of its expression has the opposite effect. Researchers hypothesized that tetraspanin 8 may have an effect on gastric cancer through activation of the ERK/MAPK and EGFR/AKT signaling pathways.^50^, ^51^ Proteomic analyses have also indicated another potential pathway of action for tetraspanin 8 in gastric cancer. Silencing of tetraspanin 8 expression led to decreased β‐catenin expression and restricted transport into the nucleus. These effects led to a decrease in the Wnt/β‐catenin pathway activity.^52^

Recently conducted studies have shown that tetraspanin 8 expression in melanomas can be associated with the invasive form of cutaneous melanoma.^53^ It has been postulated that tetraspanin 8 mediates invasiveness by preventing the aggregation of β1‐integrins and reducing the phosphorylation of ILK kinase without affecting the expression level of β1‐integrin.^54^ In addition, the exosomal form of tetraspanin 8 may contribute to the invasiveness of melanoma cells.^55^ In rare cases, the expression of the tumor suppressor protein p53 can be lost in melanoma cells. Experimental data suggest that this loss results in increased tetraspanin 8 expression and a tetraspanin 8‐dependent increase in melanoma cell invasion.^56^ Worth mentioning, tetraspanin 8 plays a role in melanoma that goes beyond intracellular functions. In vitro experiments showed that tetraspanin 8‐overexpressing melanoma cells activated pro‐MMP‐9, leading to the degradation of type IV collagen and colonization of the dermis. This was accompanied by an increase in the levels of the active form of MMP‐3 and by a decreased TIMP‐3 protein levels and these changes in protein concentrations enhance pro‐MMP‐9 activation.^57^

In turn, in hepatocellular carcinoma, it is assumed that tetraspanin 8 promotes invasion and formation of metastases without affecting the proliferation rate. Recent studies have also indicated a positive correlation between tetraspanin 8 expression and shorter overall survival. Analysis of tetraspanin 8 expression in correlation with clinical data suggested that tetraspanin 8 may be an independent predictor of postoperative survival and recurrence‐free survival. Furthermore, the effect of tetraspanin 8 in hepatocellular carcinoma may be related to a direct increase in tetraspanin 8 expression by ADAM12m.^58^ A possible role of tetraspanin 8 as a mediator of invasiveness has also been postulated and AEG‐1 protein has been suggested to regulate tetraspanin 8 expression in hepatocellular carcinomas.^59^

Conducted studies also indicate that tetraspanin 8 expression is increased in colorectal cancer compared with matched healthy control tissue.^60^ In addition, studies have indicated that tetraspanin 8 may play a role in promoting the EMT, which is dependent on LSD‐1 protein.^61^ The second proposed mechanism of action of tetraspanin 8 in colorectal cancer is its positive effect on the expression of stem cell markers *SOX2* and *ALDH1*. This is achieved through a direct interaction with β‐catenin, which increases its expression and this interaction may involve a positive feedback loop; β‐catenin binds to the promoter of the *TSPAN8* gene and enhances its transcription.^62^

It has been demonstrated that increased expression of tetraspanin 8 in breast cancer stem cells promotes the expression of stem cell markers such as NANOG, OCT4, and ALDH1. Additionally, it is associated with resistance to treatment, possibly through interaction with PTCH1.^63^ Tetraspanin 8 expression is also elevated in cancer cells exhibiting mesenchymal features, particularly in the triple‐negative breast cancer subtype. This upregulation correlates with breast cancer progression, including shorter survival and higher disease stages. Studies have suggested a potential role of tetraspanin 8 in breast cancer progression through interaction with STAT‐3 and its ability to translocate from the cytoplasm to the nucleus upon stimulation with EGF.^63^, ^64^

Moreover, the role of tetraspanin 8 in breast cancer progression has been demonstrated in in vivo experiments, using MCF‐7 and MDA‐MB‐231 breast cell line xenografts. Injection of antibodies directed against tetraspanin 8 resulted in tumor reduction.^63^ Additionally, an inhibition of cancer progression was observed in spheroids derived from breast cancer patients.^64^

Recently, Fan et al. showed that a subpopulation of cancer‐associated fibroblasts (CAF) expresses tetraspanin 8, contributing to chemoresistance and decreased overall survival in breast cancer patients.^65^ Mechanistically, tetraspanin 8‐positive CAFs express IL‐6 and IL‐8, which are associated with chemoresistance. In vivo experiments using mice bearing human xenografts further demonstrated the impact of tetraspanin blockade in sensitizing breast cancer to chemotherapy.^65^ These findings suggest the potential for anti‐tetraspanin treatment in breast cancer therapy.

Investigation of the function of tetraspanin 8 in pancreatic adenocarcinoma suggests that this protein is overexpressed in cancer stem cells. This may lead to an increased ability to migrate and form distant metastases.^66^ Examination of exosomes from pancreatic adenocarcinoma cells revealed the presence of tetraspanin 8 in these vesicles. It has been showed that exosomal tetraspanin 8 interracts with integrins and proteases, thereby participating in remodeling of the extracellular matrix. In addition, it is probably involved in the promotion of the EMT.^54^, ^67^ Therefore, exosomal tetraspanin 8 is thought to promote tumor invasion and stimulate angiogenesis in all cancers.^68^ Consequently, a study depicting the expression of tetraspanin 8 in clear cell renal cell carcinoma suggested its increased expression in cancer cells compared with healthy cells. It may be associated with a higher degree of malignancy and shorter overall survival (Table 1).^69^

**TABLE 1:** Summary of the roles of tetraspanin 6, 7, and 8 in cancer initiation and progression.

Type of cancer	Effect of tetraspanin on cancer	Potential mechanism of action	References
Tetraspanin 6			
Colorectal cancer	Inhibition of tetraspanin 6 expression increases the incidence and size of adenoma in mouse model	Loss of TSPAN 6 activates EGFR‐ dependent pathway. Syntein 1 can bind TSPAN 6 and decrease level of its active form May take part in the regulation of EMT	[39]
Lung cancer	Tumor suppressor protein	Tumor suppression by interaction with EGFR and inhibition of EGFR‐RAS‐ERK pathway	[40]
Pancreatic cancer	Tumor suppressor protein		[40]
Breast cancer	Enhances the immune response against the tumor	Enhanced invasion of B lymphocytes by increasing concentration of LXR receptor ligands in EVs	[41]
Tetraspanin 7			
Multiple myeloma	Overexpression of TSPAN 7 reduces tumor size in mouse model	Unknown	[42]
Bladder cancer	Decreased expression of TSPAN 7 in clinical cases	Induction of apoptosis by interaction with BAX proteins and caspase 3. Inhibition of cancer growth via PTEN/PI3K/AKT pathway	[44]
Osteosarcoma	Inhibition of TSPAN 7 expression inhibits tumor growth	Interaction of tetraspanin 7 with integrin β1, leads to activation of the FAK‐Src‐Ras‐ERK1/2 signaling cascade. Regulation of EMT	[3]
Tetraspanin 8			
Malignant gliomas	Suppression of TSPAN 8 expression inhibits growth and migration of cells and increases efficacy of temozolomide therapy	Activation of FAK kinase. Formation of complexes with α3 integrin and Rictor protein. Influence of aforementioned complex on cytoskeleton via AKT and Cα‐kinase. Reduction of TSPAN 8 expression by AMP kinase	[45, 46, 47]
Gastric cancer	Increased expression in cancer cells. Positive corelation with clinicopathological features	Activation of the ERK/MAPK, EGFR/AKT, and Wnt/β‐catenin signaling pathways	[48, 49, 50, 51]
Melanomas	TSPAN mediates the invasiveness of melanoma	TSPAN 8 prevents aggregation of β1‐integrins. Reduction in the phosphorylation of ILK kinase. Activation of pro‐MMP‐9 protein. Increase in TSPAN 8 expression by p53 protein loss	[52, 53, 54, 55, 56]
Hepatocellular carcinoma	Promotion of invasiveness and formation of metastasis	ADAM12m and AEG‐1 proteins regulate the expression of TSPAN 8	57, 58
Colorectal cancer	Promotion of EMT. Increase in the population of cancer stem cells	TSPAN8 promotion of EMT may be dependent on the LSD‐1 expression. Increased expression of SOX‐2 and ALDH‐1 stem cells markers by direct interaction with β‐catenin	[59, 61]
Breast cancer	Association with treatment resistance and mesenchymal features of cells.	TSPAN 8 increases the expression of NANOG, OCT 4, and ALDH1 by interaction with PTCH1 protein. Potential role of the interaction with STAT‐3 in disease progression	[62, 63, 64]
Pancreatic adenocarcinoma	Promotion of invasiveness and formation of metastasis. Remodeling of extracellular matrix. Stimulation of angiogenesis. Promotion of EMT	Exosomal form of TSPAN 8 interacts with integrins and proteases in remodeling of extracellular matrix	[54, 67, 68, 69]
Clear cell renal cell carcinoma	Increased expression of TSPAN8 in cancer cells	Unknown	[70]

Currently, monoclonal antibodies are used to inhibit the activation of signaling pathways dependent on tetraspanins, including tetraspanin 8.^70^ The observed biological response is related to the main mechanisms of carcinogenesis process and can be considered a potential target for therapy. Activation of the ERK kinase, also known as p42/44 MAP kinase which belongs to the MAPK kinases family, is a fundamental signaling pathway in carcinogenesis. Mitogen‐activated protein kinases (MAPKs) promote many cellular processes that are dysregulated in carcinogenesis.^71^, ^72^ In CRC organoids with reduced TSPAN6 expression, there was an increase in ERK1/2 phosphorylation.^39^ This association was confirmed in a mouse model of CRC.^39^ Previous studies have also investigated the role of tetraspanin 7 in the activation of the ERK signaling pathway in human osteosarcoma cells MG‐63, U2OS and HOS. In these studies, inhibition of tetraspanin 7 expression led to a concomitant reduction in the migratory capacity of osteosarcoma cells. Shao et al. suggested that tetraspanin 7 interacts with integrin β1, leading to activation of the EMT via the FAK‐Src‐Ras‐ERK1/2‐dependent signaling pathway.^3^ Studies performed on the human gastric cancer cell lines MGC‐803, AGS, MKN‐28, and BGC‐823 have shown that TSPAN8 is involved in the activation of the ERK/MAPK signaling pathway. Inhibition of TSPAN8 by siRNA led to reduced migration and proliferation of cancer cells, accompanied by a decrease in pERK levels.^50^

Additionally, the potential of tetraspanin 8 to activate the mTOR signaling pathway represents a promising target for the development of anticancer therapies for gliomas.^45^, ^73^ Ongoing clinical trials are investigating whether the use of new mTOR pathway inhibitors such as temsirolimus and everolimus is beneficial in GBM therapy and may suggest high potential of this protein inhibition.^74^ Moreover, previously published in vitro studies have demonstrated the role of tetraspanin 8 in promoting human glioma cell lines migration through the formation of complexes with α3 integrin and Rictor protein.^47^ This complex stimulates activation of the mTORC2 signaling pathway and phosphorylation of Akt (Ser475). Both these pathways are critical for the promotion of GBM cell migration and progression. In addition, studies using siRNA for *TSPAN8* and α3‐integrin have shown that the involvement of tetraspanin 8 is not direct but rather determines the formation of an active complex.^47^

Interestingly, tetraspanin 7 (TSPAN7) is currently known for its anticancer effect as it inhibits the PI3K/Akt‐dependent signaling pathway in bladder cancer. Yu et al. showed that tetraspanin 7 affected the activation of Bax, caspase‐3, and PTEN proteins while inhibiting the activity of Bcl‐2, p‐PI3K, and p‐Akt.^44^

## 7 EPITHELIAL‐MESENCHYMAL TRANSITION

EMT is a process in which epithelial cells acquire the ability to move. This process is also applicable to cancer cells. As a result of EMT, cancer cells adopt a migratory and aggressive phenotype that favors metastasis. The loss of cell–cell interactions and increase in cell‐matrix interactions are complex and multifactorial processes that require the involvement of different molecules. Uncontrolled proliferation can result from a lack of cell–cell interactions. The motility of cancer cells is crucial for invasion and the development of metastases. During the epithelial to mesenchymal transition (EMT), epithelial markers such as E‐cadherin are lost, and mesenchymal markers such as N‐cadherin and vimentin appear.^75^ The transcription factors SNAIL1, SNAIL2, TWIST1, and ZEB1 are involved in the transformation of the epithelial to mesenchymal phenotype.

The shift in phenotype requires changes in cellular metabolism and cytoskeleton restructuring. Some of these processes may be regulated by the interaction of different tetraspanins with integrins. Several studies have described the role of tetraspanins in the initiation and regulation of EMT. Tetraspanin 6 binds to EGFR (epidermal growth factor receptor) and thereby inhibits RAS activation. It has also been shown that inactivation of tetraspanin 6 induces EMT and inhibits cell migration both in vitro and in vivo.^39^ In non‐small cell lung cancer (NSCLC) cells, tetraspanin 7 induces EMT, leading to increased cell migration and proliferation. Studies in mice model have shown that NSCLC tumors formed by cells overexpressing tetraspanin 7 exhibit decreased levels of N‐cadherin and increased expression of E‐cadherin.^76^, ^77^ Moreover, a correlation between increased tetraspanin 7 levels and lymph node metastases has been previously observed^75^ and high expression of tetraspanin 7 is connected with poor patients overall survival. Additionally, increased expression of tetraspanin 7 has been detected in primary osteosarcoma tumors and osteosarcoma cell lines. Most importantly, tetraspanin 7 promotes osteosarcoma cell invasion and metastasis by inducing EMT and activating the FAK‐Src‐Ras‐ERK1/2 signaling pathway.^3^

Another issue concerns the EGFR‐AKT‐TSPAN8‐STAT3 signaling pathway upregulation in various human cancers and its association with their aggressive phenotype and poor prognosis.^76^ Tetraspanin 8 mediates the EMT by upregulating E‐cadherin and downregulating Twist, P120‐catenin and β‐catenin.^78^ Tetraspanins regulate actin dynamics and are involved in EMT. Integrins are thought to contribute to cancer progression through their interactions with tetraspanins.

## 8 TETRASPANINS AND EXOSOMES

Exosomes are nanovesicles that originate from multivesicular bodies (MVBs) and contain mRNAs and miRNAs, among others. They are also enriched in tetraspanins and tetraspanin‐related proteins.^79^ It has been shown that these EVs contribute to both autocrine regulation of tumor cell proliferation and paracrine communication between different cells in the tumor microenvironment. Exosome function is determined by the cell type from which it originates. Exosomes derived from antigen‐presenting cells trigger an immune response. However, when originating from the tumor, inhibit this response.^4^, ^5^ Tetraspanins are components of exosomes that influence their function.^80^ Current evidence shows that tumor‐derived exosomes play an important role in the interaction between tumors and other somatic cells. The target selectivity of exosomes is based on tetraspanin‐related exosomal receptors that bind ligands on target cells and exosomal tetraspanins activate signaling pathways in the target cells.^81^ Tetraspanin 6 significantly affected exosome production in HEK293 cells. Proteomics used to identify proteins present in EVs of the NSCLC cell line 393P (non‐metastatic line) and 344SQ cell line (metastatic line) showed that tetraspanin 8 levels were higher in EVs produced by the 344SQ cells.^5^, ^17^ Moreover, plasma exosomes containing tetraspanin 8 may serve as potential markers for lung cancer, especially for the non‐small cell subtype.^81^, ^82^, ^83^ Furthermore, in vitro studies have shown that overexpression of tetraspanin 8 increases the invasive ability of NSCLC cells.^82^

## 9 TETRASPANIN 8 IN TARGETED THERAPIES

Tetraspanins, in particular 6, 7, and 8, have been identified as potential therapeutic targets. Elevated levels of tetraspanin 8 have been observed in the serum of GBM patients^84^ and in in vitro cultures of human glioblastoma cells U‐87 MG and U251 MG.^45^ Previous studies performed on glioma cell lines U251 MG and U‐87 MG have shown that deactivation of the mTOR signaling pathway by activation of AMPK (AMP‐activated protein kinase) with GSK621 leads to degradation of tetraspanin 8.^45^ In addition, the use of shRNA to inhibit AMPK resulted in a reversible effect with activation of the mTOR pathway and a reduction in tetraspanin 8 degradation. As a result, glioma cell lines U‐87 MG and U251 MG were resensitized to TMZ, and a decrease in tetraspanin 8 expression levels was observed. This finding is particularly important in the context of GBM therapy, in which TMZ, an anthracycline derivative, is a leading chemotherapeutic agent and drug‐resistant phenotypes can develop during treatment.^85^ In addition, published research suggests a possible oncogenic role for tetraspanin 8 in GBM. The role of tetraspanin 8 in the progression of GBM is suggested by (i) its higher expression in tumor specimen than in nearby normal tissue, (ii) its positive correlation with the degree of histologic malignancy (grading), and (iii) its positive effect on the proliferation and migration of U‐87 MG and U251 MG cells.^45^, ^47^ These data suggest a potential therapeutic effect that may be related to the inhibition of tetraspanin 8 activity or the reduction in its expression in glioblastoma cells.

In CRC, an elevated tetraspanin 8 level is associated with poor prognosis and the development of metastases.^36^ Tetraspanin 8 is thought to facilitate colorectal cancer metastasis by promoting cell migration and interaction with adhesion proteins. In addition, elevated levels of tetraspanin 8 in blood serum may have diagnostic significance in the screening for colorectal cancer. This is supported by a study in which the mRNA levels in the blood (serum/plasma) of 64 colorectal cancer patients and 64 healthy individuals were analyzed.^85^ Furthermore, in vivo studies using a mouse model of human CRC xenografts derived from HT‐29 cells have shown that antibodies directed against tetraspanin 8 can inhibit cell migration and angiogenesis.^86^ In another study, administration of lutetium‐radiolabeled [177Lu] anti‐tetraspanin 8 monoclonal antibodies DOTA‐Ts29.2 resulted in a significant reduction in tumor size compared to controls.^36^ These results suggest that a targeted therapeutic approach focusing on tetraspanin 8 may provide grounds for further potential clinical implications. This therapy appears to be particularly effective in the case of tumors with higher tetraspanin 8 expression, such as colorectal cancer,^36^ glioblastoma,^45^, ^47^ melanoma,^55^ hepatocellular carcinoma,^87^ ovarian cancer,^58^, ^88^ and gastric cancer.^50^, ^73^ It is important to note that cancer cells have significantly higher tetraspanin 8 expression than normal cells do.

Additionally, the expression of tetraspanin 8 has been shown to be present in up to 52% of patients with epithelial ovarian cancer.^73^ In vitro studies using human ovarian cancer cells SNU8, SNU251, and SK‐OV3 have shown that targeting tetraspanin 8 with monoclonal antibodies decreased cancer cell motility and invasiveness.^73^ Additionally, in an in vivo model, monoclonal antibodies directed against tetraspanin 8 led to a reduction in metastasis.^58^

Moreover, studies performed in a mouse model of human hepatocellular carcinoma have shown that tetraspanin 8 promotes angiogenesis, migration and metastasis of cancer cells. However, these processes were inhibited in an in vivo mouse model into which knocked‐out cancer cells were administered.^58^ At the biomechanistic level, Akiel et al. found that reducing the expression of tetraspanin 8 led to a decrease in the expression of N‐cadherin and an increase in E‐cadherin expression. However, no changes were observed in the case of proteins involved in EMT. These results suggest that tetraspanin 8 interacts with membrane proteins rather than playing a role in EMT.^58^ To date, studies have demonstrated the efficacy of monoclonal antibodies against tetraspanin 8 in hepatocellular carcinoma which strongly suggest anti‐cancer potential of this treatment.^58^

**FIGURE 2:**
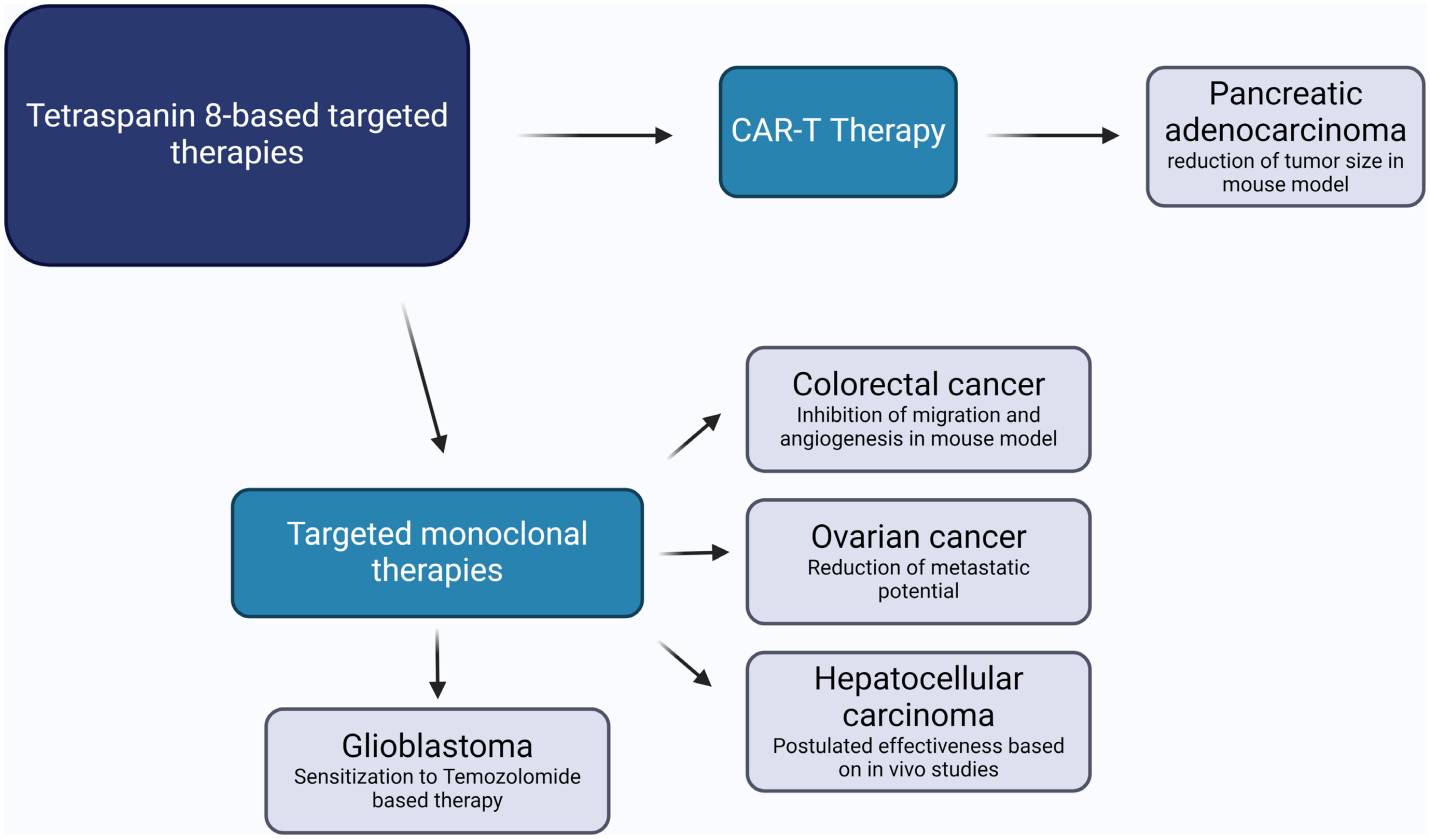
Summary of current advances in tetraspanin 8‐based therapies.

Chimeric antigen receptor (CAR) T‐cells are one of the newest strategy based on immune mechanism to fight with hematopoietic cancers like B‐cell lymphoma. In case of solid tumors, at this moment, this therapy is under clinical trials.^58^ Recently conducted research revealed that tetraspanin 8 is a protein that can be used in the development of CAR‐T therapy for pancreatic adenocarcinoma, together with new identified targets CD66c and CD318. In vivo studies have shown that this therapy gives a significant reduction of tumors induced in mice with patient‐derived pancreatic cancer cells. However, Schaefer and colleagues also observed the intestinal toxicity of this therapy and suggested the possibility of reducing it by modifying the CAR method itself. Moreover, this is one of the most promising therapeutic idea of the last decades in this highly aggressive tumor.^89^ Recently, CAR‐T therapy was developed also for tetraspanin 7,^90^ suggesting possible future applications. However, no examples of its application have been presented yet (Figure 2).

## 10 CONCLUSIONS

In this review, tetraspanins 6, 7, and 8 are presented as proteins with wide‐ranging functions in both physiological and pathological processes, especially in the development and progression of various types of neoplasms. Remarkably, tetraspanins 6 and 7 have potential anticancer effects, and loss of their expression promotes tumor development. In contrast to other tetraspanins, tetraspanin 8 evidently promotes cancer progression. The network of interactions with tetraspanins discussed in this review suggests a potential use of these proteins in cancer therapies. Recent studies have shown that monoclonal antibodies targeting tetraspanin 8 can be used in ovarian cancer, and moreover in CAR‐T therapy for pancreatic cancer. The growing number of reports and research results concerning the molecular mechanisms of action of tetraspanins 6, 7 and 8 makes them a promising area of research with a focus on new anticancer therapies. However, the exact function of tetraspanins 6, 7, and 8 in cellular machinery is still not yet fully understood, and detailed roles remain to be defined.

## AUTHOR CONTRIBUTIONS

**Monika Mrozowska:** Conceptualization (equal); project administration (equal); supervision (equal); writing – original draft (equal). **Tomasz Górnicki:** Project administration (equal); visualization (equal); writing – original draft (equal). **Mateusz Olbromski:** Writing – review and editing (equal). **Aleksandra Izabela Partyńska:** Writing – review and editing (equal). **Piotr Dzięgiel:** Project administration (equal); writing – review and editing (equal). **Agnieszka Rusak:** Project administration (equal); supervision (equal); writing – original draft (equal).

## FUNDING INFORMATION

Not applicable.

## CONFLICT OF INTEREST STATEMENT

The authors declare no conflict of interest.

## Data Availability

Not applicable.

## References

[1] Charrin S , Jouannet S , Boucheix C , Rubinstein E . Tetraspanins at a glance. J Cell Sci. 2014;127(17):3641‐3648. doi:10.1242/jcs.154906 25128561

[2] Termini CM , Gillette JM . Tetraspanins function as regulators of cellular signaling. Front Cell Dev Biol. 2017;5:1‐14. doi:10.3389/fcell.2017.00034 28428953 PMC5382171

[3] Shao S , Piao L , Guo L , et al. Tetraspanin 7 promotes osteosarcoma cell invasion and metastasis by inducing EMT and activating the FAK‐Src‐Ras‐ERK1/2 signaling pathway. Cancer Cell Int. 2022;22(1):1‐16. doi:10.1186/s12935-022-02591-1 35524311 PMC9074275

[4] Berditchevski F , Odintsova E . Tetraspanins as regulators of protein trafficking. Traffic. 2007;8(2):89‐96. doi:10.1111/j.1600-0854.2006.00515.x 17181773

[5] Garcia‐Mayea Y , Mir C , Carballo L , Sánchez‐García A , Bataller M , LLeonart ME . TSPAN1, a novel Tetraspanin member highly involved in carcinogenesis and chemoresistance. Biochim Biophys Acta Rev Cancer. 2022;1877(1):188674. doi:10.1016/j.bbcan.2021.188674 34979155

[6] Yáñez‐Mó M , Barreiro O , Gordon‐Alonso M , Sala‐Valdés M , Sánchez‐Madrid F . Tetraspanin‐enriched microdomains: a functional unit in cell plasma membranes. Trends Cell Biol. 2009;19(9):434‐446. doi:10.1016/j.tcb.2009.06.004 19709882

[7] Kovalenko OV , Metcalf DG , DeGrado WF , Hemler ME . Structural organization and interactions of transmembrane domains in tetraspanin proteins. BMC Struct Biol. 2005;5:1‐20. doi:10.1186/1472-6807-5-11 15985154 PMC1190194

[8] Termini CM , Cotter ML , Marjon KD , Buranda T , Lidke KA , Gillette JM . The membrane scaffold CD82 regulates cell adhesion by altering Α4 integrin stability and molecular density. Mol Biol Cell. 2014;25(10):1560‐1573. doi:10.1091/mbc.E13-11-0660 24623721 PMC4019488

[9] Lee SA , Suh Y , Lee S , et al. Functional expression of dopamine D2 receptor is regulated by tetraspanin 7‐mediated postendocytic trafficking. FASEB J. 2017;31(6):2301‐2313. doi:10.1096/fj.201600755RR 28223337

[10] Jiang X , Zhang J , Huang Y . Tetraspanins in cell migration. Cell Adhes Migr. 2015;9(5):406‐415. doi:10.1080/19336918.2015.1005465 PMC4955372 26091149

[11] Jumper J , Evans R , Pritzel A , et al. Highly accurate protein structure prediction with AlphaFold. Nature. 2021;596:583‐589. 34265844 10.1038/s41586-021-03819-2 PMC8371605

[12] Stipp CS , Kolesnikova TV , Hemler ME . Functional domains in tetraspanin proteins. Trends Biochem Sci. 2003;28(2):106‐112. doi:10.1016/S0968-0004(02)00014-2 12575999

[13] Wright MD , Tomlinson MG . The ins and outs of the transmembrane 4 superfamily. Immunol Today. 1994;15(12):588‐594. doi:10.1016/0167-5699(94)90222-4 7531445

[14] Maecker HT , Todd SC , Levy S . The tetraspanin superfamily: molecular facilitators. FASEB J. 1997;11(6):428‐442. 9194523

[15] Susa KJ , Kruse AC , Blacklow SC . Tetraspanins: structure, dynamics, and principles of partner‐protein recognition. Trends Cell Biol. 2023;34(6):509‐522. doi:10.1016/j.tcb.2023.09.003 37783654 PMC10980598

[16] Seigneuret M , Delaguillaumie A , Lagaudrière‐Gesbert C , Conjeaud H . Structure of the tetraspanin main extracellular domain: a partially conserved fold with a structurally variable domain insertion. J Biol Chem. 2001;276(43):40055‐40064. doi:10.1074/jbc.M105557200 11483611

[17] Umeda R , Satouh Y , Takemoto M , et al. Structural insights into tetraspanin CD9 function. Nat Commun. 2020;11(1):1‐7. doi:10.1038/s41467-020-15459-7 32231207 PMC7105497

[18] Hemler ME . Tetraspanin functions and associated microdomains. Nat Rev Mol Cell Biol. 2005;6(10):801‐811. doi:10.1038/nrm1736 16314869

[19] Wang F , Vandepoele K , Van Lijsebettens M . Tetraspanin genes in plants. Plant Sci. 2012;190:9‐15. doi:10.1016/j.plantsci.2012.03.005 22608515

[20] Yunta M , Lazo PA . Tetraspanin proteins as organisers of membrane microdomains and signalling complexes. Cell Signal. 2003;15(6):559‐564. doi:10.1016/S0898-6568(02)00147-X 12681443

[21] Perot BP , Ménager MM . Tetraspanin 7 and its closest paralog tetraspanin 6: membrane organizers with key functions in brain development, viral infection, innate immunity, diabetes and cancer. Med Microbiol Immunol. 2020;209(4):427‐436. doi:10.1007/s00430-020-00681-3 32468130

[22] Maeda K , Matsuhashi S , Hori K , et al. Cloning and characterization of a novel human gene, TM4SF6, encoding a protein belonging to the transmembrane 4 superfamily, and mapped to Xq22. Genomics. 1998;52(2):240‐242. doi:10.1006/geno.1998.5415 9782095

[23] McLaughlin KA , Richardson CC , Ravishankar A , et al. Identification of tetraspanin‐7 as a target of autoantibodies in type 1 diabetes. Diabetes. 2016;65(6):1690‐1698. doi:10.2337/db15-1058 26953162

[24] Takagi S , Fujikawa K , Imai T , et al. Identification of a highly specific surface marker of T‐cell acute lymphoblastic leukemia and neuroblastoma as a new member of the transmembrane 4 superfamily. Int J Cancer. 1995;61:706‐715. doi:10.1002/ijc.2910610519 7768645

[25] Zemni R , Bienvenu T , Vinet MC , et al. A new gene involved in X‐linked mental retardation identified by analysis of an X;2 balanced translocation. Nat Genet. 2000;24(2):167‐170. doi:10.1038/72829 10655063

[26] Hedera P , Alvarado D , Beydoun A , Fink JK . Novel mental retardation‐epilepsy syndrome linked to Xp21.1‐p11.4. Ann Neurol. 2002;51(1):45‐50. doi:10.1002/ana.10051 11782983

[27] Bassani S , Passafaro M . TSPAN7: a new player in excitatory synapse maturation and function. BioArchitecture. 2012;2(3):95‐97. doi:10.4161/bioa.20829 22880149 PMC3414387

[28] Pang S , Luo Z , Dong W , et al. Integrin Β1/FAK/SRC signal pathway is involved in autism spectrum disorder in Tspan7 knockout rats. Life Sci Alliance. 2023;6(3):1‐17. doi:10.26508/lsa.202201616 PMC9768919 36625203

[29] Murru L , Vezzoli E , Longatti A , et al. Pharmacological modulation of AMPAR rescues intellectual disability‐like phenotype in Tm4sf2−/y mice. Cereb Cortex. 2017;27(11):5369‐5384. doi:10.1093/cercor/bhx221 28968657 PMC5939231

[30] Kwon JO , Lee YD , Kim H , et al. Tetraspanin 7 regulates sealing zone formation and the bone‐resorbing activity of osteoclasts. Biochem Biophys Res Commun. 2016;477(4):1078‐1084. doi:10.1016/j.bbrc.2016.07.046 27416754

[31] Guix FX , Sannerud R , Berditchevski F , et al. Tetraspanin 6: a pivotal protein of the multiple vesicular body determining exosome release and lysosomal degradation of amyloid precursor protein fragments. Mol Neurodegener. 2017;12(1):1‐21. doi:10.1186/s13024-017-0165-0 28279219 PMC5345265

[32] McLaughlin KA , Tombs MA , Christie MR . Autoimmunity to Tetraspanin‐7 in type 1 diabetes. Med Microbiol Immunol. 2020;209(4):437‐445. doi:10.1007/s00430-020-00674-2 32314012 PMC7395010

[33] Eugster A , Kraus G , Lidzba V , Müller D , Jolink M , Ziegler A . Cytoplasmic ends of tetraspanin 7 harbour epitopes recognised by autoantibodies in type 1 diabetes. Diabetologia. 2019;62:805‐810. 30789994 10.1007/s00125-019-4832-2

[34] Helassa N . TSPAN‐7 as a key regulator of glucose‐stimulated Ca2^+^ influx and insulin secretion. J Physiol. 2021;599(6):1733‐1734. doi:10.1113/JP281053 33347614

[35] Hald J , Galbo T , Rescan C , et al. Pancreatic islet and progenitor cell surface markers with cell sorting potential. Diabetologia. 2012;55(1):154‐165. doi:10.1007/s00125-011-2295-1 21947380

[36] McLaughlin K , Acreman S , Nawaz S , et al. Loss of tetraspanin‐7 expression reduces pancreatic β‐cell exocytosis Ca2^+^ sensitivity but has limited effect on systemic metabolism. Diabet Med. 2022;39(12):e14984. doi:10.1111/dme.14984 36264270 PMC9828109

[37] Hirukawa T , Wu Q , Sawada K , et al. Kidney diseases enhance expression of tetraspanin‐8: a possible protective effect against tubular injury. Nephron Extra. 2014;4(1):70‐81. doi:10.1159/000362451 24926311 PMC4036207

[38] Zöller M . Tetraspanins: push and pull in suppressing and promoting metastasis. Nat Rev Cancer. 2009;9(1):40‐55. doi:10.1038/nrc2543 19078974

[39] Zhu H , Wu Y , Zheng W , Lu S . CO‐029 is overexpressed in gastric cancer and mediates the effects of EGF on gastric cancer cell proliferation and invasion. Int J Mol Med. 2015;35(3):798‐802. doi:10.3892/ijmm.2015.2069 25592989

[40] Andrijes R , Hejmadi RK , Pugh M , et al. Tetraspanin 6 is a regulator of carcinogenesis in colorectal cancer. Proc Natl Acad Sci USA. 2021;118(39):e2011411118. doi:10.1073/PNAS.2011411118 34521767 PMC8488650

[41] Humbert PO , Pryjda TZ , Pranjic B , et al. TSPAN6 is a suppressor of Ras‐driven cancer. Oncogene. 2022;41(14):2095‐2105. doi:10.1038/S41388-022-02223-Y 35184157 PMC8975741

[42] Molostvov G , Gachechiladze M , Shaaban AM , et al. Tspan6 stimulates the chemoattractive potential of breast cancer cells for B cells in an EV‐ and LXR‐dependent manner. Cell Rep. 2023;42(3):112207. doi:10.1016/j.celrep.2023.112207 36867531

[43] Cheong CM , Chow AWS , Fitter S , et al. Tetraspanin 7 (TSPAN7) expression is upregulated in multiple myeloma patients and inhibits myeloma tumour development in vivo. Exp Cell Res. 2015;332(1):24‐38. doi:10.1016/J.YEXCR.2015.01.006 25637218

[44] Olar A , Goodman LD , Wani KM , et al. A gene expression signature predicts recurrence‐free survival in meningioma. Oncotarget. 2018;9(22):16087‐16098. doi:10.18632/ONCOTARGET.24498 29662628 PMC5882319

[45] Yu X , Li S , Pang M , et al. TSPAN7 exerts anti‐tumor effects in bladder cancer through the PTEN/PI3K/AKT pathway. Front Oncol. 2021;10:10. doi:10.3389/FONC.2020.613869 PMC7821430 33489923

[46] Jiang H , Liu W , Zhan SK , et al. GSK621 targets glioma cells via activating AMP‐activated protein kinase signalings. PLoS One. 2016;11(8):e0161017. doi:10.1371/JOURNAL.PONE.0161017 27532105 PMC4988667

[47] Pan SJ , Wu YB , Cai S , et al. Over‐expression of tetraspanin 8 in malignant glioma regulates tumor cell progression. Biochem Biophys Res Commun. 2015;458(3):476‐482. doi:10.1016/J.BBRC.2015.01.128 25680464

[48] Pan SJ , Zhan SK , Pan YX , et al. Tetraspanin 8‐rictor‐integrin Α3 complex is required for glioma cell migration. Int J Mol Sci. 2015;16(3):5363‐5374. doi:10.3390/IJMS16035363 25761241 PMC4394480

[49] Mottaghi‐Dastjerdi N , Soltany‐Rezaee‐Rad M , Sepehrizadeh Z , Roshandel G , Ebrahimifard F , Setayesh N . Identification of novel genes involved in gastric carcinogenesis by suppression subtractive hybridization. Hum Exp Toxicol. 2015;34(1):3‐11. doi:10.1177/0960327114532386 24812152

[50] Zhang L , Xu Y , Cai E , et al. TSPAN8 regulates EGFR/AKT pathway to enhance metastasis in gastric cancer. Mol Biol Rep. 2023;50:7955‐7965. doi:10.1007/s11033-023-08662-4 37535246

[51] Wei L , Li Y , Suo Z . TSPAN8 promotes gastric cancer growth and metastasis via ERK MAPK pathway. Int J Clin Exp Med. 2015;8(6):8599‐8607. 26309511 PMC4537951

[52] Li L , Yang D , Cui D , et al. Quantitative proteomics analysis of the role of tetraspanin‐8 in the drug resistance of gastric cancer. Int J Oncol. 2018;52(2):473‐484. doi:10.3892/IJO.2017.4231 29345284

[53] Berthier‐Vergnes O , Kharbili ME , De La Fouchardière A , et al. Gene expression profiles of human melanoma cells with different invasive potential reveal TSPAN8 as a novel mediator of invasion. Br J Cancer. 2011;104(1):155‐165. doi:10.1038/SJ.BJC.6605994 21081927 PMC3039798

[54] El Kharbili M , Robert C , Witkowski T , et al. Tetraspanin 8 is a novel regulator of ILK‐driven Β1 integrin adhesion and signaling in invasive melanoma cells. Oncotarget. 2017;8(10):17140‐17155. doi:10.18632/ONCOTARGET.15084 28188308 PMC5370029

[55] Zhao K , Erb U , Hackert T , Zöller M , Yue S . Distorted leukocyte migration, angiogenesis, wound repair and metastasis in Tspan8 and Tspan8/CD151 double knockout mice indicate complementary activities of Tspan8 and CD51. Biochim Biophys Acta, Mol Cell Res. 2018;1865(2):379‐391. doi:10.1016/j.bbamcr.2017.11.007 29138006

[56] Agaësse G , Barbollat‐Boutrand L , El Kharbili M , Berthier‐Vergnes O , Masse I . P53 targets TSPAN8 to prevent invasion in melanoma cells. Oncogene. 2017;6(4):e309. doi:10.1038/ONCSIS.2017.11 PMC5520488 28368391

[57] El Kharbili M , Cario M , Béchetoille N , et al. Tspan8 drives melanoma dermal invasion by promoting ProMMP‐9 activation and basement membrane proteolysis in a keratinocyte‐dependent manner. Cancers (Basel). 2020;12(5):1297. doi:10.3390/CANCERS12051297 32455575 PMC7281247

[58] Fang T , Lin J , Wang Y , et al. Tetraspanin‐8 promotes hepatocellular carcinoma metastasis by increasing ADAM12m expression. Oncotarget. 2016;7(26):40630‐40643. doi:10.18632/ONCOTARGET.9769 27270327 PMC5130032

[59] Akiel MA , Santhekadur PK , Mendoza RG , Siddiq A , Fisher PB , Sarkar D . Tetraspanin 8 mediates AEG‐1‐induced invasion and metastasis in hepatocellular carcinoma cells. FEBS Lett. 2016;590(16):2700‐2708. doi:10.1002/1873-3468.12268 27339400 PMC4992437

[60] Maisonial‐Besset A , Witkowski T , Navarro‐Teulon I , et al. Tetraspanin 8 (TSPAN 8) as a potential target for Radioimmunotherapy of colorectal cancer. Oncotarget. 2017;8(13):22034‐22047. doi:10.18632/oncotarget.15787 28423546 PMC5400644

[61] Zhang HS , Liu HY , Zhou Z , Sun HL , Liu MY . TSPAN8 promotes colorectal cancer cell growth and migration in LSD1‐dependent manner. Life Sci. 2020;241:241. doi:10.1016/J.LFS.2019.117114 31790687

[62] Zhan Z , Zhong L , Feng M , Guo Y . A positive tetraspanin 8 (TSPAN8)/β‐catenin regulatory loop enhances the stemness of colorectal cancer cells. Med Sci Monit. 2019;25:9594‐9601. doi:10.12659/MSM.919749 31838484 PMC6929559

[63] Zhu R , Gires O , Zhu L , et al. TSPAN8 promotes cancer cell stemness via activation of sonic hedgehog signaling. Nature Commun. 2019;10(1):2863. doi:10.1038/s41467-019-10739-3 31253779 PMC6599078

[64] Sandoval K , Weiss WA . Nuclear tetraspanin 8 promotes breast cancer progression. Cell Res. 2022;32(6):511‐512. doi:10.1038/S41422-022-00657-3 35418219 PMC9160050

[65] Fan G , Yu B , Tang L , Zhu R , et al. TSPAN8^+^ myofibroblastic cancer–associated fibroblasts promote chemoresistance in patients with breast cancer. Sci Transl Med. 2024;16:eadj5705. doi:10.1126/scitranslmed.adj5705 38569015

[66] Lu X , An L , Fan G , et al. EGFR signaling promotes nuclear translocation of plasma membrane protein TSPAN8 to enhance tumor progression via STAT3‐mediated transcription. Cell Res. 2022;32(4):359‐374. doi:10.1038/S41422-022-00628-8 35197608 PMC8975831

[67] Wang H , Rana S , Giese N , Büchler MW , Zöller M . Tspan8, CD44v6 and Alpha6beta4 are biomarkers of migrating pancreatic cancer‐initiating cells. Int J Cancer. 2013;133(2):416‐426. doi:10.1002/IJC.28044 23338841

[68] Yue S , Mu W , Erb U , Zöller M . The tetraspanins CD151 and Tspan8 are essential exosome components for the crosstalk between cancer initiating cells and their surrounding. Oncotarget. 2015;6(4):2366‐2384. doi:10.18632/ONCOTARGET.2958 25544774 PMC4385857

[69] Zhao K , Wang Z , Hackert T , Pitzer C , Zöller M . Tspan8 and Tspan8/CD151 knockout mice unravel the contribution of tumor and host exosomes to tumor progression. J Exp Clin Cancer Res. 2018;37(1):312. doi:10.1186/s13046-018-0961-6 30541597 PMC6292129

[70] Tang Y , Xie J , Chen Y , et al. Tspan8 is highly expressed in clear cell renal cell carcinoma and indicates poor prognosis. Ann Clin Lab Sci. 2020;50(5):638‐644. 33067209

[71] Vences‐Catalán F , Levy S . Immune targeting of tetraspanins involved in cell invasion and metastasis. Front Immunol. 2018;9:1‐7. doi:10.3389/fimmu.2018.01277 29946318 PMC6006414

[72] Sugiura R , Satoh R , Takasaki T . Erk: a double‐edged sword in cancer. Erk‐dependent apoptosis as a potential therapeutic strategy for cancer. Cells. 2021;10(10):2509. doi:10.3390/cells10102509 34685488 PMC8533760

[73] Smalley I , Smalley KSM . VIEWS ERK inhibition: a new front in the war against MAPK pathway–Driven Cancers? Cancer Discov. 2018;8(2):140‐142. doi:10.1158/2159-8290.CD-17-1355 29431672

[74] Heo K , Lee S . TSPAN8 as a novel emerging therapeutic target in cancer for monoclonal antibody therapy. Biomolecules. 2020;10(3):388. doi:10.3390/BIOM10030388 32138170 PMC7175299

[75] Wu W , Klockow JL , Zhang M , et al. Glioblastoma multiforme (GBM): An overview of current therapies and mechanisms of resistance. Pharmacol Res. 2021;171:105780. doi:10.1016/j.phrs.2021.105780 34302977 PMC8384724

[76] Kim KK , Kugler MC , Wolters PJ , et al. Alveolar epithelial cell mesenchymal transition develops in vivo during pulmonary fibrosis and is regulated by the extracellular matrix. Proc Natl Acad Sci USA. 2006;103(35):13180‐13185. doi:10.1073/pnas.0605669103 16924102 PMC1551904

[77] Wang X , Lin M , Zhao J , Zhu S , Xu M , Zhou X . TSPAN7 promotes the migration and proliferation of lung cancer cells via epithelial‐to‐mesenchymal transition. Onco Targets Ther. 2018;13(11):8815‐8822. doi:10.2147/OTT.S167902 PMC6300375 30588007

[78] Yao S , Gan C , Wang T , Zhang Q , Zhang M , Cheng H . High ALDH2 expression is associated with better prognosis in patients with gastric cancer. Am J Cancer Res. 2022;12(12):5425‐5439. 36628272 PMC9827082

[79] Voglstaetter M , Thomsen AR , Nouvel J , et al. Tspan8 is expressed in breast cancer and regulates E‐cadherin/catenin signalling and metastasis accompanied by increased circulating extracellular vesicles. J Pathol. 2019;248(4):421‐437. doi:10.1002/path.5281 30982971 PMC6771825

[80] Zhang L , Zhang S , Yao J , et al. Microenvironment‐induced PTEN loss by exosomal microRNA primes brain metastasis outgrowth. Nature. 2015;527(7576):100‐104. doi:10.1038/nature15376 26479035 PMC4819404

[81] Hemler ME . Tetraspanin proteins promote multiple cancer stages. Nat Rev Cancer. 2014;14(1):49‐60. doi:10.1038/nrc3640 24505619

[82] Rana S , Yue S , Stadel D , Zöller M . Toward tailored exosomes: the exosomal tetraspanin web contributes to target cell selection. Int J Biochem Cell Biol. 2012;44(9):1574‐1584. doi:10.1016/j.biocel.2012.06.018 22728313

[83] Sandfeld‐Paulsen B , Jakobsen KR , Bæk R , et al. Exosomal proteins as diagnostic biomarkers in lung cancer. J Thorac Oncol. 2016;11(10):1701‐1710. doi:10.1016/J.JTHO.2016.05.034 27343445

[84] Liu Y , Fan J , Xu T , et al. Extracellular vesicle tetraspanin‐8 level predicts distant metastasis in non‐small cell lung cancer after concurrent chemoradiation. Sci Adv. 2020;6(11):eaaz6162. doi:10.1126/SCIADV.AAZ6162 32195353 PMC7065889

[85] Tunckale T et al. Investigation of serum E‐cadherin, VEGF121, Survivin, Tenascin C and Tetraspanin 8 levels in patients with glioblastoma. Bratisl Lek Listy. 2023;124(4):304‐308. doi:10.4149/BLL_2023_046 36598325

[86] Rodia MT , Ugolini G , Mattei G , et al. Systematic large‐scale meta‐analysis identifies a panel of two MRNAs as blood biomarkers for colorectal cancer detection. Oncotarget. 2016;7(21):30295‐30306. doi:10.18632/oncotarget.8108 26993598 PMC5058681

[87] Ailane N , Greco C , Zhu Y , et al. Effect of an anti‐human Co‐029/Tspan8 mouse monoclonal antibody on tumor growth in a nude mouse model. Front Physiol. 2014;5:1‐9. doi:10.3389/fphys.2014.00364 25285080 PMC4168815

[88] Park CS , Kim T‐K , Kim HG , et al. Therapeutic targeting of Tetraspanin8 in epithelial ovarian cancer invasion and metastasis. Oncogene. 2016;35(34):4540‐4548. doi:10.1038/onc.2015.520 26804173

[89] Schäfer D , Tomiuk S , Küster LN , et al. Identification of CD318, TSPAN8 and CD66c as target candidates for CAR T cell based immunotherapy of pancreatic adenocarcinoma. Nat Commun. 2021;12(1):1‐18. doi:10.1038/s41467-021-21774-4 33674603 PMC7935963

[90] Pieper T , Roth KDR , Glaser V , et al. Generation of chimeric antigen receptors against tetraspanin 7. Cells. 2023;12(11):1‐15. doi:10.3390/cells12111453 PMC10252682 37296574

